# Effect of organic loading rates on the performance of membrane bioreactor for wastewater treatment behaviours, fouling, and economic cost

**DOI:** 10.1038/s41598-023-42876-7

**Published:** 2023-09-20

**Authors:** Aly Al-Sayed, Gamal K. Hassan, Mona T. Al-Shemy, Fatma A. El-gohary

**Affiliations:** 1 Water Pollution Research Department, National Research Centre, 33El-Bohouth St. (Former El-Tahrir St.), Dokki, P.O. 12622, Giza, Egypt; 2 Cellulose and Paper Department, National Research Centre, 33El-Bohouth St. (Former El-Tahrir St.), Dokki, P.O. 12622, Giza, Egypt

**Keywords:** Environmental sciences, Environmental chemistry, Pollution remediation

## Abstract

Although submerged membrane bioreactor (MBR) are widely used in treating municipal wastewater and recovery of potential resources, membrane operational parameters and membrane fouling control remain debated issues. In this study, the treatment of municipal wastewater by MBR at high-biomass sludge (MLSS (g/L) ranging from 5.4 g/L to 16.1 g/L) was assessed at an organic loading rates (OLRs) ranging from 0.86 to 3.7 kg COD/m^3^d. The correlation between trans-membrane pressure and total fouling resistance was thoroughly investigated in this study. According to the findings, greater OLRs of 0.86 to 3.7 kg COD/m^3^d caused a decrease in COD, BOD, and NH_4_–N removal efficiency, and higher OLRs of 3.7 kg COD/m^3^d resulted in a higher increase in total fouling resistance (R_*t*_). The economic study of using the MBR system proved that for a designed flow rate of 20 m^3^/d, the payback period from using the treated wastewater will be 7.98 years, which confirms the economic benefits of using this MBR for treating municipal wastewater. In general, understanding the challenges facing the efficiency of MBR would improve its performance and, consequently, the sustainability of wastewater reclamation.

## Introduction

The problem of water scarcity in Egypt has prompted a large number of scholars to look into alternative solutions in order to delay the catastrophic effects of this calamity on the lives of the people and the economy of the country^1,2^. The use of treated wastewater is one of the possible solutions. Nevertheless, there are numerous issues with this, including an increase in the complexity and detrimental effect of wastewater generated by industries and discharged into the sewer system without pretreatment, a situation which brought about stringent water policies. Using biological wastewater treatment methods, including the conventional activated sludge (CAS) method, the majority of those challenges can be handled. Regardless, these processes are characterized by low selectivity for plenty of contaminants, like microbes and some organic contaminants^3,4^. Many problems with CAS can be resolved using the membrane bioreactor (MBR), an alternate treatment approach with a smaller footprint, higher quality effluent, and less sludge generation. Hence, MBRs are being employed more frequently than CAS processes. The main factors causing the production of high-quality recycled water by MBRs are the nearly complete retention of high molecular substances, bacteria, and suspended particles by membrane filtration. Thus, as the last stage of treatment, it separates solids from liquids far more effectively than the secondary settler in a CAS process^5^.

However, membrane filtration cannot help as much with phosphorus and nitrogen removals as per biological processes are primarily responsible for reducing nutrients (phosphorus and nitrogen). In fact, the intensive aeration and prolonged solid retention times in a conventional MBR system may make nutrient removal less effective^6,7^. Still, difficulties with membrane fouling during the treatment of the activated sludge have delayed the development of MBRs. Hence, recent studies on MBR-based domicile wastewater treatment systems have concentrated on ways to manage membrane fouling^8^. Among these are membrane surface modification, high cross-flow velocity use, chemical or operational condition optimization, and hydrodynamic cleaning^9,10^.

It is however, worth mentioning that prevalent commercial usage of MBRs is limited because of membrane fouling. Fouling phenomena on the membrane surface and inside the pores decrease long-term flux stability, requiring membrane cleaning, which raises the overall cost. In addition, if cleaning is ineffective at recovering enough flux, membrane replacement is also an option^11,12^. Due to the complexity of the phenomenon of membrane fouling, it is still difficult for scientists working in this area to forecast the fouling behavior^13^. Consequently, the rising maintenance costs due to operation as a result of fouling is one of the most significant downsides of MBR, which limits its broad adoption^14^.

Therefore, there have been numerous attempts to manage all the parameters involved in the design and operation of MBRs including: operating conditions, feed and biomass parameters, and membrane and module characteristics. As a model, it was already demonstrated that OLR and F/M caused a significant impact on the microbe-linked features like biomass growth and extracellular production, which in turn could produce either decrease or increase of membrane fouling^15,16^.

Due to developed cake layer resistance at the membrane surface, Wu et al. and Xia et al. reported substantial membrane fouling at a higher OLR^14,17^. Although numerous studies have been conducted on various occasions to investigate membrane fouling, with an emphasis on membrane types, plant configuration, permeate quality, etc.^18,19^, just a few of them have focused on the biological operation used in membrane filtration processes. Furthermore, numerous studies have shown that controlling the fluctuation of the influent organic loads' variable is likely to retain biomass concentration^20,21^.

Additionally, in order to treat municipal wastewater, Rosenberger et al.^20^ developed a hollow fiber MBR. In this system, OLRs were variable depending on the operation conditions, decreasing to 0.07 kg COD/kg MLSS/d, mixed liquor suspended solids (MLSS) concentration was adjusted between 18 and 20 g/L, and volumetric loading rates were set between 1.1 and 1.7 kg COD/m^3^d. Overall, the process was fully consistent, with a strong ability to remove organic materials and total nitrogen. Wagner and Rosenwinkel^21^ discovered that after a year of operation, when comparing the sludge production in an MBR pilot plant to the CAS, by diverging the loading rates between 0.04 and 0.2 kg COD/kg MLSS/d without removing any sludge, the MLSS concentrations increased from 2 to 18 g L^−1^, and the sludge outgrowth was less than in the CAS but remained the same.

Previous studies have shown that sludge ages from 50 to 100 days can significantly reduce sludge output, MBRs have this advantage over other wastewater purification systems since they function best at high sludge ages^22,23^. However, there is no absolute sludge age value because it depends on a combination of factors, including the system's design, feed characteristics, and operation conditions. In contrast to the bulk of systems described in the literature where membrane fouling experiments were conducted, the most recent research works were completed at a high sludge age (> 200 days). Otherwise, since actual systems depend on the feed characteristics (flow rate and concentration of the wastewater treatment plant), they cannot operate at stable OLRs. Vo et al.^24^ found that when high-strength tannery wastewater was treated in a lab-scale MBR for 280 days with an OLR of 1.3 and 2.6 kg COD/m^3^d and a sludge retention time (SRT) of 30 days, it accomplished COD removal capacities of 78 ± 19% and 89 ± 2%, respectively. Pollice et al.^25^ discovered that the system of a 6 L lab-scale MBR with hollow fiber membranes perfectly cooperated with the adjustment of different volumetric loads (0.8 and 1.7 g COD/L d) and operated for more than 100 days without sludge discharge. At a low OLR of 0.12 g COD/g TSS/ d, the equilibrium was also manageable with both loads. The system was ultimately simple to implement and had a quick startup with little sludge production. Low OLRs are thus obtained in the operation while operating at high or full sludge retention times, which definitely alter biomass conditions^25,26^.

Therefore, the current work focuses on the functionality of a lab-scale flat sheet submerged membrane bioreactor (MBR) for microfiltration of synthetic wastewater mixed with municipal wastewater. Additionally, it aims to ascertain how the variation of the applied organic load impacts the development, performance, and fouling of the biomass in the MBR system to develop a techno-economic strategy for using this technology to treat municipal wastewater in Egypt.

## Methods

### Experimental set-up and operation

**Figure 1:**
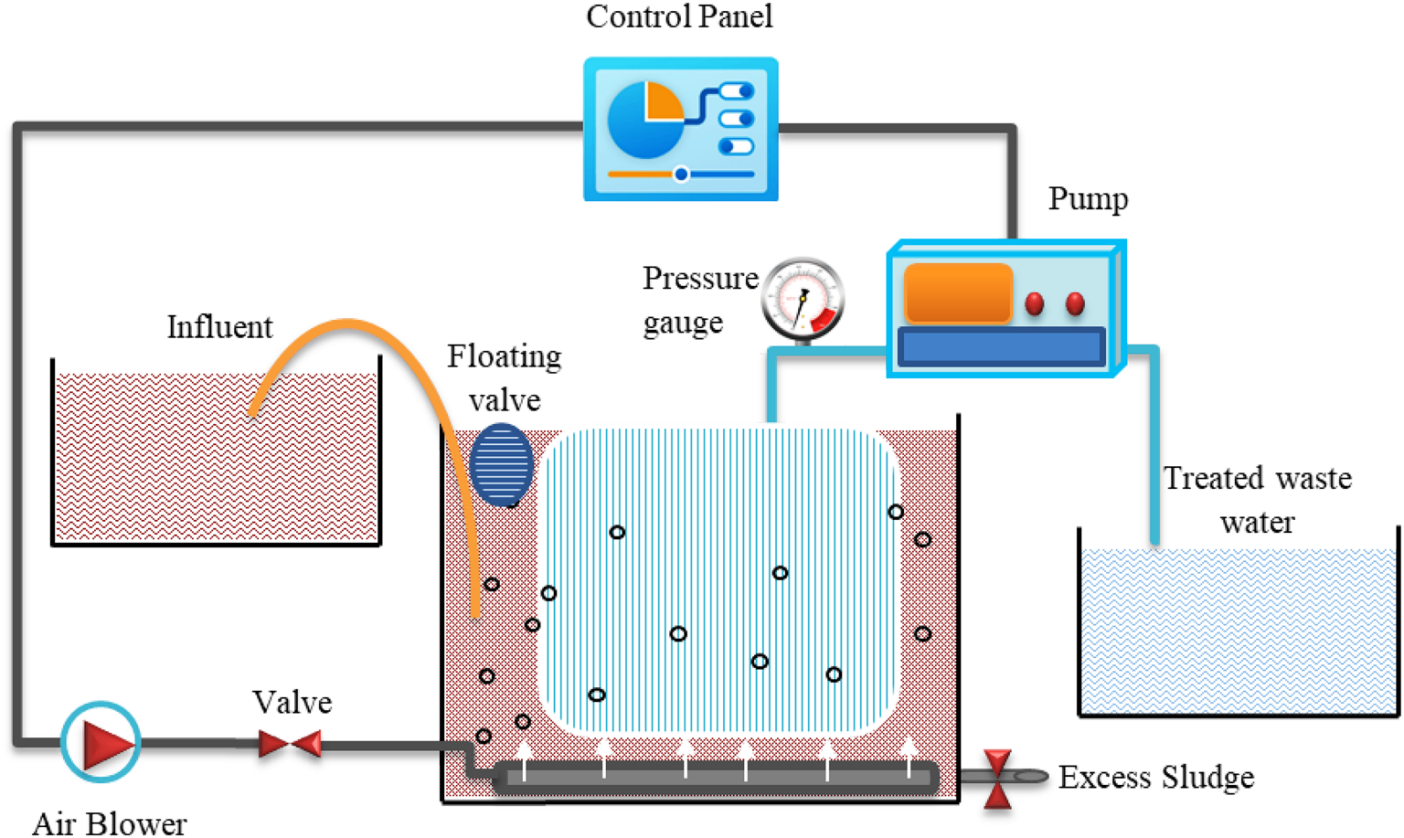
Schematic diagram of lab scale MBR treatment system.

Figure 1 displays a schematic representation of the MBR system. For operation at an HRT of 7 h, the permeate flux was held constant at 11.4 L/h m^2^. The different OLR values of 0.86, 1.8, and 3.7 kg COD/m^3^d were adjusted using synthetic wastewater. TMP was continuously monitored with an electronic pressure gauge for the propensity to foul using a peristaltic pump operating in a 10 min on/2 min off mode. Daily measurements of the permeate flow rate were taken throughout the operation to guarantee continuous flux, and TMP was also being watched for membrane fouling.

The MBR system comprised Perspex tank of 23 cm in width, 40 cm in length, and 10 cm in height, with an effective volume of 8 L. The Perspex tank contained a single flat sheet microfiltration membrane (membrane module-SINAP, Shanghai) made of polyvinylidene difluoride (PVDF) with a pore size of 0.1 m, an effective membrane area of 0.1 m^2^, a width of 22 cm, a length of 32 cm, and a thickness of 0.6 cm. At the bottom of the MBR tank, an air diffuser that provided oxygen was placed. In this reactor, dissolved oxygen (DO) concentration was kept at a level greater than 4 mg/L. To maintain the operating capacity of 8 L, an electric floating valve with a feed pump connection was installed in the MBR tank. With daily continuous sludge drainage, the SRT was maintained constant for 30 days throughout the process.

The MBR had penetration and relaxation durations of 10 and 2 min, respectively. A level controller at the top of the reactor managed the intermittent 1.14 L/h pumping of wastewater into it. The TMP value recorded by the vacuum gauge was used to determine the membrane fouling of the MBR. After each cycle, or whenever the vacuum meter index reached the value of 0.4 bars, the membrane module was cleaned using physical and chemical techniques. Solutions of 0.2% sodium hypochlorite and 1% citric acid were used for chemical cleaning. Each MBR had a DO concentration limit of 4 mg/L or higher. The experiment's temperature was kept between 23 and 28 °C.

### Feed wastewater

In order to run the MBRs at a consistent influent hydraulic load and different organic loads, the influent concentration was controlled using synthetic wastewater. Table 1 displays the wastewater's composition. Three distinct organic loads were investigated to characterize the behavior of different operational factors. Chemical oxygen demand (COD) average values of about 250, 500, and 950 mg/L were obtained for the lowest, middle, and maximum compositions, respectively, to prepare the synthetic wastewater. Molasses was used as the carbon source, and K_2_HPO_4_ and NHCl_4_ were used as the phosphorous and nitrogen sources, respectively, in the synthetic water.

**Table 1:** The three distinct OLRs obtained by changing the composition of the synthetic wastewater.

Component	Concentration (mg/L)		
	Low load	Medium load	High load
COD (Molasses + municipal wastewater)	230–290	480–561	930–1050
Ammonia (NHCl_4_)	18–23	19–25	22–44
TP (K_2_HPO_4_)	0.9–2	0.9–4.7	2.2–5.5
OLR (kg COD/m^3^d)	0.86	1.8	3.7

### Analytical methods

By observing changes in the microbiological, chemical, and physical features of the influent and infiltrates (effluents), the sludge quality, and the membrane fouling, the performance of the three MBRs was assessed.

#### Physico-chemical analysis

Mixed liquor volatile suspended solids (MLVSS), mixed liquor suspended solids (MLSS), total suspended solids (TSS), total phosphorus (TP), total nitrogen (TN), ammonium nitrogen (NH_4_–N), biological oxygen demand (BOD), and chemical oxygen demand (COD) were determined in accordance with Standard Methods for the Examination of Water and Wastewater^27^.

Samples of mixed liquor (ML) containing soluble microbial products (SMP) were taken, promptly cooled to 4 °C, and then analyzed within two hours. Although SMP contains very complex chemical compounds, proteins and polysaccharides were utilized to quantify and better characterize it because they make up a significant portion of it, as described in the literature^28^. The untreated ML was centrifuged at 12,000 *g* for 15 min to represent the soluble fraction (SMP). The quantities of protein and carbohydrates were measured in the supernatant. The methods of Lowry et al.^29^ and Dubois et al.^30^ were used to measure the calorimetric concentrations of supernatant protein and carbohydrates, respectively.

### Calculations of membrane resistance fraction

The permeate flux (J) in L/h.m^2^ was quantitatively determined employing Eq. (1),

1 $${\text{J}} = \frac{{\text{Q}}}{{A_{m} }}$$

here Q is the permeate flow rate (L/h) evaluated by measuring the collected effluent volume versus time, and A_m_ is the membrane surface area (m^2^).

The total membrane resistance was calculated according to Eq. (2)^31^,

2 $$J = \frac{\Delta p}{{\mu .R_{t} }}$$

here ΔP is the Trans membrane pressure (N/m^2^), μ is the effluent viscosity (N.s/m^2^),

3 $${\text{R}}_{t} = {\text{R}}_{m} + {\text{R}}_{c} + {\text{R}}_{f}$$

here R_*m*_ is the initial membrane resistance, (R_f_) the total organic and inorganic fouling resistance, (R_c_) the sludge layer resistance coating membrane surface during filtration. R_m_ was determined by filtrating deionized water using the new membrane. In this case the sum of R_f_ and R_c_ equals zero and as a consequence R_t_ = R_m_. The value of R_f_ was determined at the end of each run after removing the sludge layer^1^.

### Superficial morphology of activated sludge

Superficial micrographs were captured via scanning electron microscopy (Quanta FEG-250) to evaluate the surface morphology of activated sludge. After each wastewater treatment cycle, the activated sludge was taken out of the batch reactor and washed with 0.1 M phosphate buffer (pH 7.4) three successive times before being held at 4 °C for 4 h. Afterward, the sludge flocs were dehydrated gradually using the ethanol series (50%, 70%, 80%, 90%, and 100%), twice rinsed with 0.1 M phosphate buffer (pH 7.4), and then allowed to dry in the air before being viewed by the microscope at 20 kV.

### Economic cost calculations

The economic cost of the MBR system fed by municipal wastewater was established for a treatment capacity of 20 m^3^/d^32^, whereas the energy of the pumps was computed from Eq. (4) to assess the operational cost in accordance with Nguyen and Yoshikawa^33^.

4 $${\text{C}}_{{{\text{energy}}}} = \left( {{\text{E}}_{{{\text{pumps}}}} /\eta } \right) \times {8}000 \times \left( {{36}00/{1}000} \right) \times {\text{energy}}\;{\text{cost}}$$

where C _energy_ is annual energy expense (USD/y), E_pumps_ is the power supply by pumps, and η is the pumps efficiencies (0.7). The chemical cleaning and electricity expenses were calculated as stated in Tawfik et al.^34^.

## Results and discussion

### Impact of OLRs on the performance of MBR

#### Removal of organic compounds and nutrients

**Figure 2:**
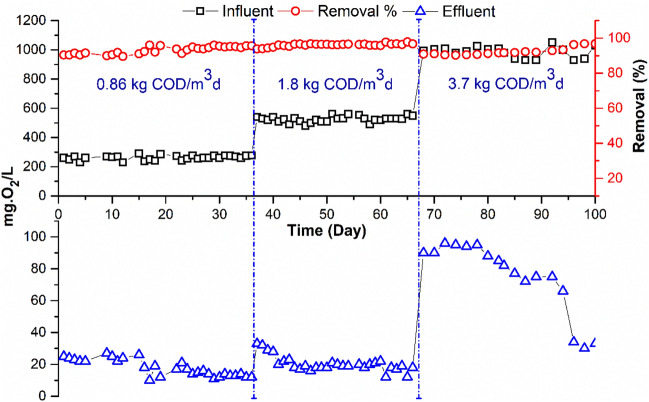
Variation of total COD in MBR influent and effluent at different OLR.

**Figure 3:**
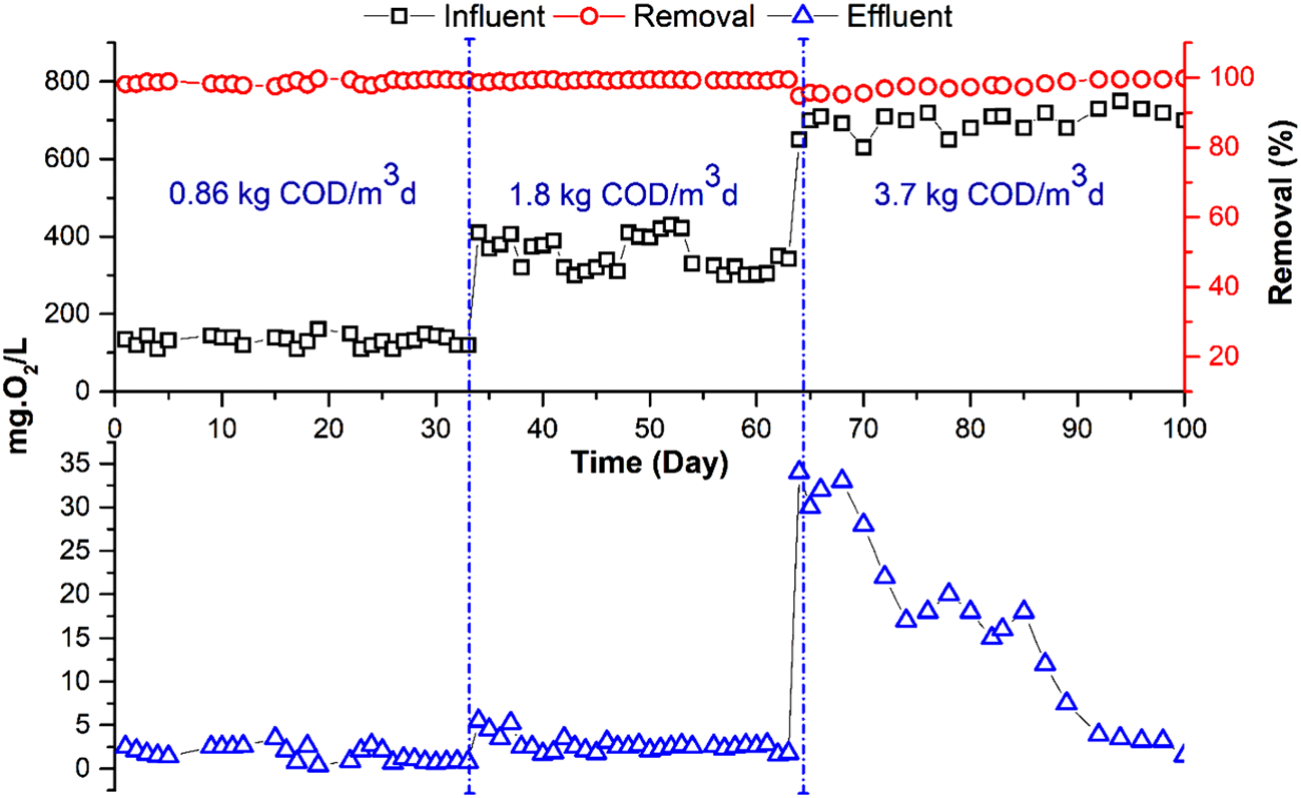
Variation of total BOD in MBR influent and effluent at different OLR.

This experiment's main objective was to determine whether employing a submerged flat sheet membrane bioreactor (MBR) to treat highly concentrated wastewater, which simulates the effluent, discharged in treatment plants receiving high OLR. Based on the results of previous experiments^1,4^, the permeate flux; HRT and SRT were kept constant at 11.4 L/h.m^2^, 7 h, and 30 days, respectively. Three different OLRs were investigated: 0.86, 1.8 and 3.7 kg COD/m^3^d. The COD value of the wastewater was adjusted to the required concentration (around 250, 500 and 950 mgO_2_/L) by adding high strength synthetic waste to the real municipal wastewater. Table 2 shows the average water quality of the influent and treated wastewater and summarizes the average efficiency of the treatment system. At an OLR of 0.86 and 1.8 kg COD/m^3^d, the MBR removed from 93.2 to 95% of the total COD and 99% of the total BOD. COD removal was reduced from 95 to 92% when increase OLR to 3.7 kg COD/m^3^d (Fig. 2). Also, BOD removal was reduced by two percentage points (Fig. 3). The COD removal efficiency was not significantly different between the two OLRs of 0.86 and 1.8 kg COD/m^3^d, but it was significantly affected at an OLR of 3.7 kg COD/m^3^d.

**Table 2:** Impact of OLR on MBR Performance.

Parameter	Unit	ORL 0.86 kg COD/m^3^d (SRT 30 d)			ORL 1.8 kg COD/m^3^d (SRT 30 d)			ORL 3.7 kg COD/m^3^d (SRT 30 d)		
		Influent	Effluent	R (%)	Influent	effluent	R (%)	Influent	effluent	R (%)
pH	–	7.40 ± .20	7.9 ± 0.16	–	7.8 ± 0.1	7.9 ± 0.2	–	7.5 ± 0.2	7.80 ± 0.2	–
TSS (105 °C)	mg/L	120.12 ± 13	0	100	112 ± 4	0.0 ± 0	100	120 ± 8	0.00 ± 0.0	100
COD tot	mg O_2_/L	252 ± 15	18 ± 5.3	93.2	532 ± 22	25 ± 5	95	970 ± 73	70 ± 25	92
BOD tot	mg O_2_/L	122.33 ± .14	1.7 ± 0.8	99	361 ± 44	2.7 ± 0.9	99	660 ± 72	20 ± 10	96
NH_4_–N	mg N/L	21.79 ± .1.3	0	100	28 ± 1.7	6.1 ± 3	78	34 ± 2.9	16 ± 4	52
NO_3_	mg N/L	0.50 ± 017	29 ± 9.8	–	0.40 ± 0.2	22 ± 8	–	0 ± 0.2	0.0 ± 0.0	–
TKN	mg N/L	38 ± .2.6	4.2 ± 3.8	86	38 ± 2.7	11 ± 3	71	44 ± 2.3	22 ± 3.3	50
TP	mg P/L	1.42 ± .016	0.9 ± 0.3	36	4.5 ± 0.7	3 ± 0.4	33	7.5 ± 0.4	5.5 ± 0.7	26

#### Impact of OLR on nitrogen species

The nitrification process, which transforms ammonia into nitrite and nitrate, is one main way that NH_4_–N is typically removed in the MBR. According to the MBR's performance results, there was a high (100–78%) conversion of NH_4_–N into NO_3_ at an OLRs of 0.86 and 1.8 kg COD/m^3^d.

**Figure 4:**
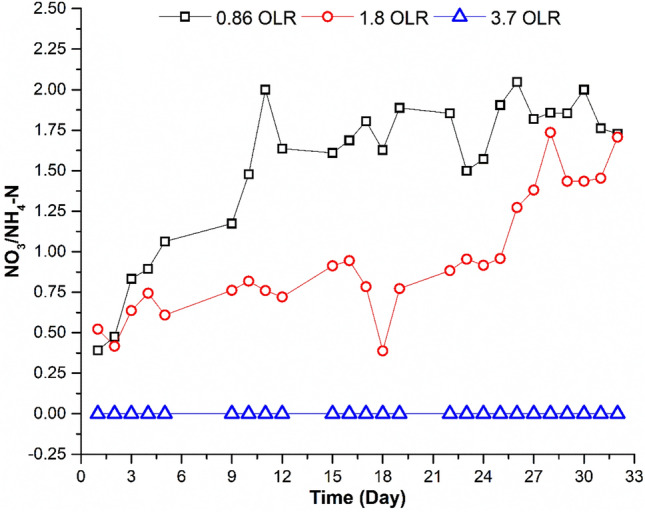
Impact of OLRs on the transformation of NH_4_–N into NO_3_.

When the OLR was increased to 3.7 kg COD/m^3^d (Table 2), the conversion of NH_4_–N into NO_3_ dropped from 78% to less than 50%. The ratio of the production of NO_3_ to the input of NH_4_–N, as illustrated in Fig. 4, can be used to describe how NH_4_–N is converted into NO_3_. The NO_3_/NH_4_–N ratio decreased as the OLR increased, (Fig. 4). Higher nitrification (higher conversion of NH_4_–N into NO_3_) is indicated by a higher NO_3_/NH_4_–N ratio. As more organic carbon is present in the reactor at higher OLRs competition between heterotrophic and autotrophic (nitrifying) organisms is expected. The elimination of inorganic nutrients was also shown to be less efficient when OLRs and F/M were higher^35,36^. It is worth mentioning that the concentration of NO_2_ was essentially within the same range of 0.01–0.03 mg/L and was not affected by changing the OLRs. This is to be expected, as the rate-limiting step in the nitrification process is the formation of nitrite.

**Figure 5:**
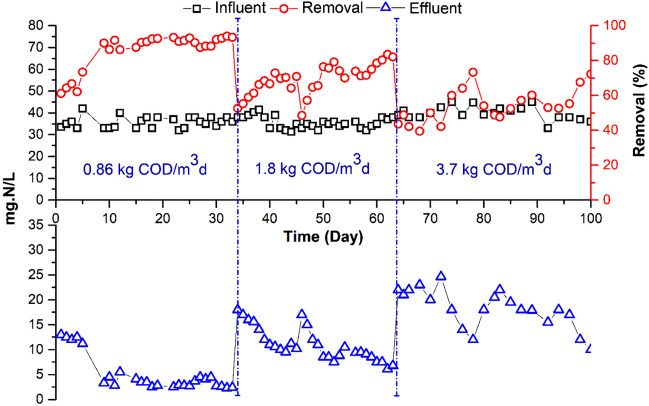
Variation of TKN in MBR influent and effluent at different OLR.

An increase of the OLR from 0.86 to 3.7 kg COD/m^3^d showed a drop in TKN removal efficiency from 86 to 53% (Fig. 5). Residual ammonia increased from 6.1 to 16 mg N/L. No nitrification has been reported at the higher OLR. At the higher organic load, average value of TKN in the effluent was 22 mg/L. This view was also supported by^37,38^. According to He et al.^39^ and Kanimozhi and Vasudevan^40^ high OLRs result in lower nitrification efficiency because of loss of ammonium through assimilation by heterotrophy. The alteration in the nitrifying bacterial population correlated with the change in nitrogen removal performance with OLR has been discussed by Xu et al.^41^. In the present study nitrate concentration of the effluent at an OLR of 0.86 and 1.8 kg COD/m^3^d was 29 to 22 mg/L, whereas no nitrification has been observed at the higher loading rate (3.7 kg COD/m^3^d). This might be due to the loss of nitrifying bacteria at the high OLR. Higher organic loads have been reported to inhibit the nitrifying bacteria, affecting negatively the nitrification process. The MBR failed to remove phosphorus effectively, as can be expected. According to Table 2, the removal effectiveness for phosphorus was 26–36%, whereas the elimination could result via adsorption onto membrane surfaces and some consumption by microorganisms when new cells develop.

#### Impact of OLR on growth of biomass

**Figure 6:**
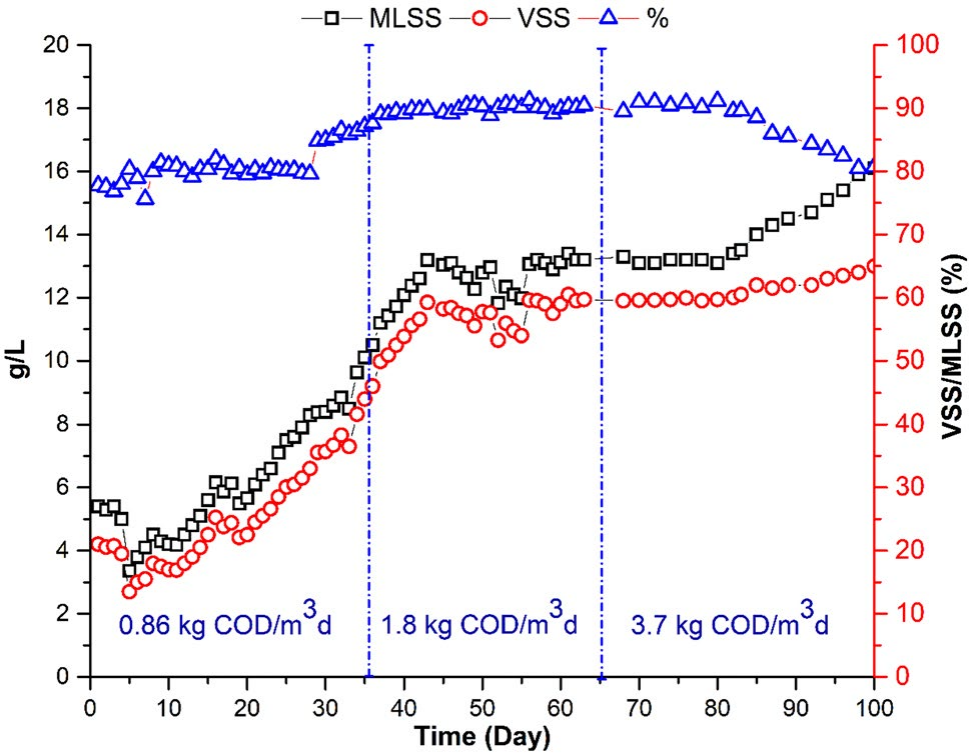
Sludge Growths (g/L) at Different OLR.

**Figure 7:**
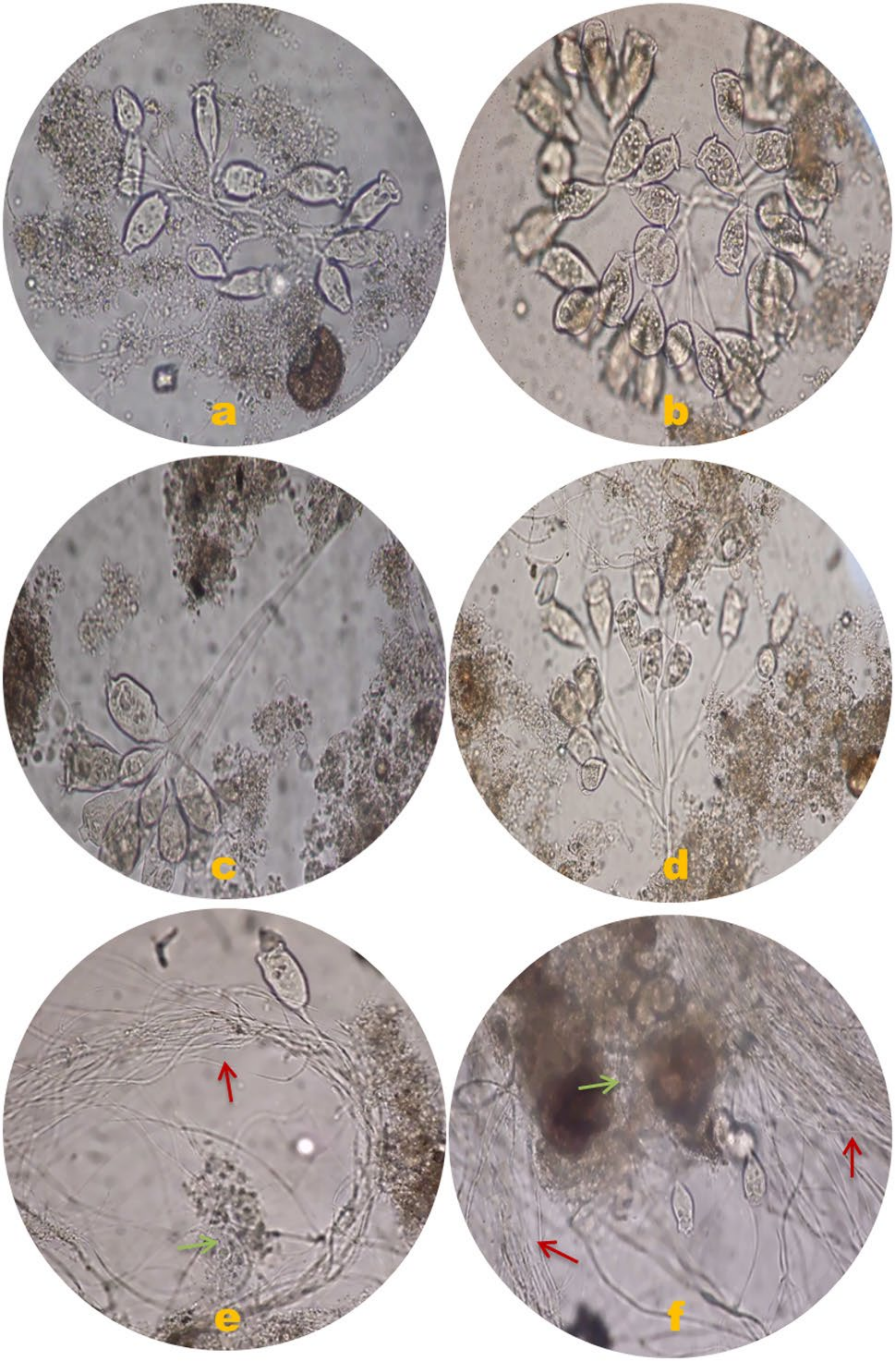
Micrographs at X20 magnification by optical microscopy of activated sludge biomass after OLRs of 0.86 (**a** and **b**), 1.8 (**c** and **d**), and 3.7 kg COD/m^3^d (**e** and **f**).

At the start-up period of the MBR operated at an OLR of 0.86 kg COD/m^3^d, MLSS was 5.4 g/L. It increased gradually until it reached steady state (13.4 g/L). The concentration remained constant till the end of the end of the second run. Increasing the OLR up to 3.7 kg COD/m^3^d, led to a sudden deterioration of the sludge quantity and quality (Fig. 6). This was followed by an increase in the MLSS up to 16.1 g/L. Microscopic examination of the biomass showed that it is dominated by filamentous organisms (Figs. 7e and f). The proliferation of filamentous bacteria causes an increase in the viscosity and hydrophobicity of the sludge. Furthermore, filamentous bacteria can improve and fix the contaminants on the membrane surface^4,42^.

Available results confirm that at a constant HRT and SRT, OLR impacts the bioreactor's biomass concentration. Domínguez et al.^43^ found that, while biomass concentrations and loading rates increased during the process, the cleaning rate declined. Further, the biomass growth rate at the highest loading rate was nearly twice that achieved in the experiment with the medium load and was five times higher than the growth rate at the lowest load. Consequently, with intermediate and higher volumetric loading rates at OLRs of 0.15 kg COD/MLVSS d, steady-state conditions were reached for the MBR, albeit more rapidly with the highest loading rate. Therefore, they recommended starting to operate MBRs at high loading rates until steady-state conditions are reached to accelerate reaching the required biomass content and minimize membrane fouling.

**Figure 8:**
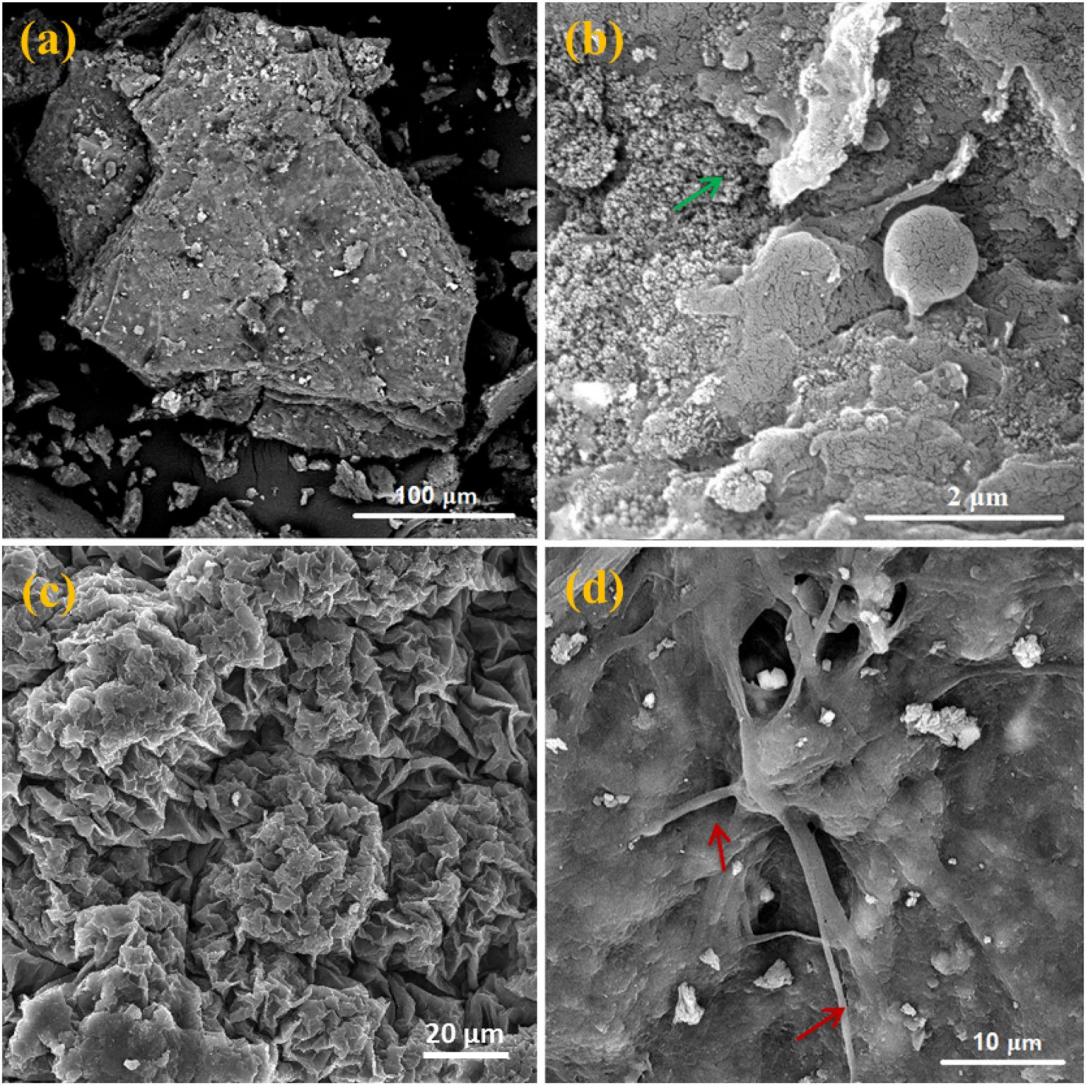
Micrographs at different magnifications by SEM imaging of activated sludge incubated in MBR (**a** and **b**) originally and after OLR 3.7 kg COD/m^3^d (**c** and **d**).

Even though the viscosity was not quantified, it was noted that at an OLR of 3.7 kg COD/m^3^d, the sludge seemed to be seized in a dense gel layer. This finding was corroborated by the optical and SEM micrographs, which revealed tiny, glowing, interconnecting globules in the activated sludge clump (Figs. 7e,f and 8). The micrographs in Fig. 7 allow us to see the discrepancy between the three operating systems. Figures 7a–d, which indicate OLR values of 0.86 and 1.8 kg COD/m^3^d, display a moist mount of big, compact, solid, well-settling flocs. On the other hand, micrographs from the OLR 3.7 kg COD/m^3^d system show open flocs and loose flocs with inter-floc filament bridging. Filamentous bacteria, which operate as a structural foundation for strong floc production, are advantageous to activated sludge settle-ability in clarifiers when present in reasonable concentration^44,45^. Therefore, for an ideal working MBR system, the growth of floc-forming bacteria and filamentous bacteria should coexist in harmony^46^. However, the presence of several physicochemical parameters and/or modifications in process conditions that indirectly affect the chemical status of the activated sludge stimulates the excessive growth of filamentous bacteria^47,48^. Depending on the type of filamentous bacteria present, filamentous overgrowth may produce either inter-floc bridging or open flocs. The first type arises when filaments emerge from the flocs into the bulk liquid, creating bridges between them and preventing isolated flocs from getting compacted (referred to by the red arrows in the Fig. 7e and f). While the second kind develops when many filaments grow inside weakly consolidated flocs, thereby trapping water within the flocs (referred to by the green arrows in the Fig. 7e and f)^47^. In light of this, the proliferation of filamentous bacteria can cause an increase in the viscosity and hydrophobicity of the sludge, which can result in the formation of various foulants and membrane fouling, as seen in the MBR system with an OLR of 3.7 kg COD/m^3^d. Contrarily, no filaments were found when MBR was run at OLR 0.86 and 1.8 kg COD/m^3^d, providing yet more evident proof of the cause of the performance disparity between the three operating systems and the impact of OLR on the membrane fouling.

SEM analysis was used to examine the morphological aspects of the activated sludge from the OLR at 3.7 kg COD/m^3^d. The bacteria in the activated sludge developed clumps of biofilms in the operating system with a variety of surface patterns, as seen in the SEM micrographs at various magnifications of different sections of the active sludge in Fig. 8. The MBR system created a biofilm with a smooth surface and few holes while running at an OLR of 3.7 kg COD/m^3^d. These openings provide water channels that let nutrients and oxygen reach the majority of the biofilm's cells. This variation and alteration in the forms created by the biofilm at an OLR of 3.7 kg COD/m^3^d may be brought on by an increase in the OLR, which improves the mass transfer of nutrients to the biofilm and causes variations in the biofilm's thickness and texture. De Kievit discovered that, as Pseudomonas *aeruginosa's* biofilm evolved, microcolonies multiplied to form structures that resembled mushrooms and stalks under conditions that restricted bacterial migration (such as glucose). However, when movement was encouraged (by glutamate and succinate), cells prevalently grew and formed a flat, regular mat^49^. These findings are consistent with what is seen in Fig. 8c and d. This is also in line with earlier findings that showed the decline performance of activated sludge under 3.7 kg COD/m^3^d OLR working conditions.

#### Resistance analysis

To assess the impact of increasing the OLR on membrane filtration characteristics, total hydraulic resistance values were calculated. The results obtained indicated that increasing the OLR from 0.86 to 3.7 kg COD/m^3^d, increased total fouling resistance (*R*_*t*_) from 1.56 × 10^12^ to 7.23 × 10^12^. Johir et al.^50^ studied the impact of OLR on membrane fouling using MBRs operating at six different OLRs ranging from 0.5 to 3.0 kg COD/m^3^d, at constant HRT and SRT of 8 h and 40 days. Their research's conclusions showed that greater OLRs (2.75–3 kg COD/m^3^d) increased fouling rates. Using two identical laboratory-scale submerged MBRs run for 162 days at an SRT of 30 days, Zhang et al.^51^ examined the impact of constant and changing influent OLRs on membrane fouling. According to reports, the MBR receiving variable loading had more substantial membrane fouling than the MBR fed with a constant OLR during the start-up phase. The MBR performed under the various OLRs, however, showed less membrane fouling after reaching a steady state than the MBR run at the constant OLR. Changes in extracellular polymeric substance (EPS) and biofloc particle size over an entire period of operation could provide a sufficient explanation for the observed occurrences. Variable loading appears to be a potential alternative operational method for preventing membrane fouling in MBRs throughout the duration of prolonged operation.

### Membrane fouling

**Figure 9:**
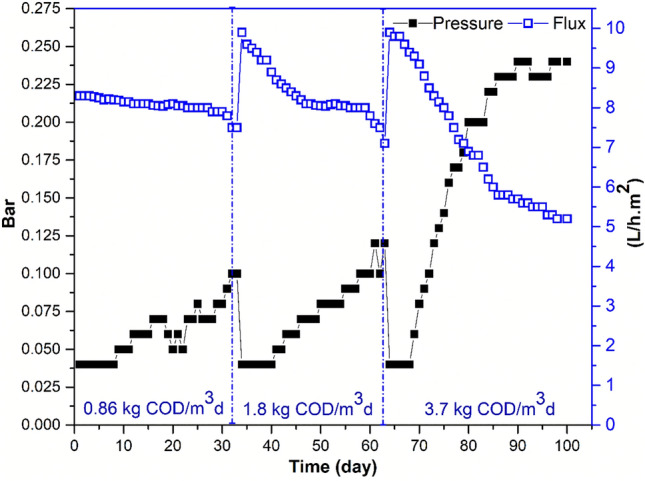
TMP and Flux Variation at different OLR (0.86, 1.8, 3.7 kg COD/m^3^d).

Transmembrane pressure (TMP) monitoring was used in the current investigation to characterize fouling that occurred during MBR operation. Figure 9 depicts the TMP variation throughout the MBR's operation for more than 90-day. Phase 1 (days 1 through 35), Phase 2 (days 35 through 66), and Phase 3 (days 66 through 100) generally correspond to the three phases. The TMP was rising with time at a fairly moderate rate during this period 0.86 kg COD/m^3^d. OLR of 3.7 kg COD/m^3^d was the only OLR used during the third phase of the MBR's operation. A rapid rise in TMP was seen throughout this phase. The findings indicate that an increase in OLR from 0.86 to 3.7 kg COD/m^3^d has caused a considerable rise in the rate of membrane fouling to the point where the air scouring approach is no longer effective in maintaining the membrane clean. This general conclusion is in agreement with earlier MBR investigations on the impact of OLR on membrane fouling^42,52–54^. There is little agreement in these researches as to the cause of the impact of the shift in OLR on membrane fouling, though. Yet, in the current study, the fast increase in the TMP observed at the higher OLR could be due to the growth in MLSS up to 14.5 g/L at day 18. Similar findings have been reported by Çiçek et al.^55^ and Chang and Kim^56^, who attributed the higher development of TMP to the higher OLR. According to Ng and Hermanowicz^57^, formation of non-flocculated micro-organisms, which continuously attach onto the membrane surface as F/M ratio increases, causes an increase in TMP. Additionally, it has been noted that the buildup of hydrophilic chemicals on the membrane surface led to increased membrane fouling^58^. In the present study, increasing OLR from 0.86 to1.8 kg COD/m^3^d and from 1.8 to 3.7 kg COD/m^3^d increased MLSS concentrations from 5.4 to 8.8 and from 8.8 to 13.4 and 13.4 to 16.1 g/L, respectively (Fig. 9). This confirms the findings of previous studies.

#### Soluble microbial products (SMP) and fouling effects

**Figure 10:**
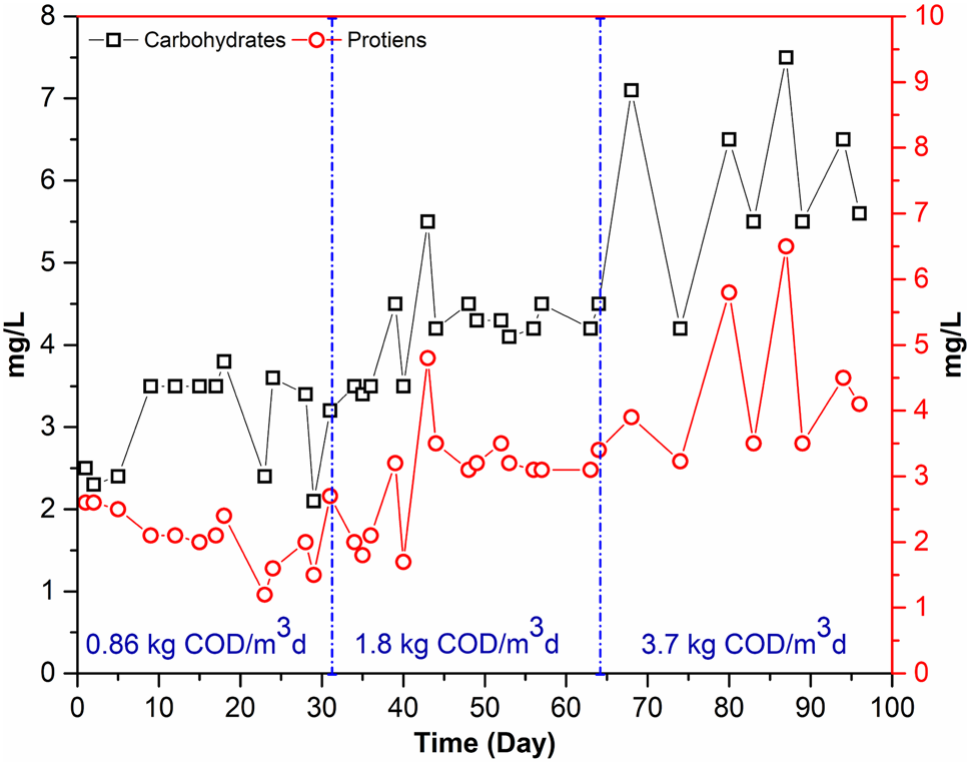
Carbohydrate and protein contents at different OLR (0.86, 1.8, 3.7 kg COD/m^3^d).

One of the key factors contributing to membrane fouling in MBRs has been identified as SMP. However, conflicting reports exist on the impact of SMP on membrane fouling. The compounds that are present in the supernatant but are absent from or only mildly present in the permeate are likely to be foulants. In the supernatant, soluble substances are released as a result of biomass action. The SMP are made up of a number of different chemicals, but the majority of them include humic acids, nucleic acids, proteins, and polysaccharides, with the first two present in much lesser amounts. Therefore, to assess the likelihood of fouling, attention was kept on the predominant species, particularly the polysaccharides and proteins in the supernatant^59^. The polysaccharide findings in the supernatant are shown in Fig. 10. This suggests that the retention of these molecules, as previously described^60,61^, was caused by the creation of a gel layer. Since increases in polysaccharide concentrations tend to make the sludge less filterable, it has been acknowledged that the soluble polysaccharide content may serve as a proxy for the degree of fouling^60,62,63^. In the supernatant, protein concentrations were almost always far lower than polysaccharide concentrations (Fig. 10), demonstrating that polysaccharides have a more significant impact on membrane fouling than proteins do, as described in the literature^64–66^. Additionally, no relationship between feed fluctuation and the requirement for mechanical cleaning was discovered, confirming that protein concentrations only slightly affect fouling.

Throughout the process, the concentrations of polysaccharides and proteins in the permeate are shown in Fig. 10. Over the course of the OLR 0.86 to 3.7 kg COD/m^3^d operations, the polysaccharide and protein concentrations ranged from 2.5–7.3 mg/L and 2–4 mg/L, respectively. The results showed that, when compared to operations with high OLR, SMP concentrations were reduced in low OLR operations. SMP concentrations are known to have a considerable impact on membrane fouling, particularly internal fouling. Regarding the mechanisms of membrane fouling, SMP result in membrane pore obstruction by adsorption of their pore walls. Additionally, the mixed liquor has poor filterability as a result of the SMP quickly building up in the MBR due to membrane rejection. Furthermore, the polysaccharide in SMP contributes more to membrane fouling than protein^67^. Fu et al.^68^ also said that the reduced EPS concentration, specifically lower polysaccharide concentrations, may be the primary cause of the improved filtration performance that was attained in the MB-MBR process as compared to traditional MBR. The faster TMP increase in OLR of 3.7 kg COD/m^3^d allowed more SMP to deposit onto the membrane surface due to a higher drag force. This helped to form the cake layer, which in turn caused more SMP to be generated by increased microbial activity or endogenous decay and cell lysis inside the sludge cake^8,69^. In addition, the cake layer generated on the membrane surface helped organic materials adhere and become adsorbent. On the other hand, the inclusion of bio-carriers significantly decreased the growth of SMP and EPS on the membrane surface, which mitigated membrane fouling^8^.

### Economic study for using MBR to treat municipal wastewater

The economic evaluation was carried out for the reclamation of municipal wastewater as shown in Table 3. The total capital expenses are 50,140 USD, but since the treatment process using MBRs is planned to be a development process to replace the conventional methods in an existing wastewater treatment facility, site leasing and building on it are not included in the capital expenses. On the other hand, the cost of the membrane unit, which includes the frame and membrane module and has a lifespan of 20 and 10 years, respectively, accounts for around 35% of overall capital expenses. While pumps for balancing, sludge, vacuum, lifting, etc., represent the second highest capital expenses by up to 30%. Prices for all capital expenditures were acquired from either the vendor or the manufacturer.

**Table 3:** Economic evaluation of the pilot-scale MBR for municipal wastewater treatment (For a flow rate of 20 m^3^/d and 7300 m^3^/y, the proposed membrane area is 51.4 m^2^).

	Capital expenses (USD)		Operational expenses (USD/y)		Economic profit	
	Item	Cost	Item	Costs	Item	Benefits
Pilot-scale MBR	Tanks for feed, permeate, and sludge	5000	Electrical consumption = 80 kw/d (0.16 USD/kWh)	4672	Cost for 1 m^3^ of recycled water	0.64 USD/m^3^
	Membrane unit (i.e., frame and module)	17,640	Sludge disposal	3.6	Cost to buy 1 m^3^ of water	1.50 USD/m^3^
	Pumps (for balancing, sludge, vacuum, lifting, etc.)	15,200	Chemicals for cleaning	2.7	Profit using MBR system	0.86 USD/m^3^
	PLC control system (transducers and instrument display for pressure, temperature, conductivity, etc.)	5000			Annually profit using MBR system	6278 (USD/y)
	Accessories (cabling, pipe, valve, flow meter, etc.)	4200				
	Others (manufacture, transport, installation, etc.)	3100				
Total	50,140		4678.3		6278 (USD/y)	
Payback period	Capital expenses/Economic profits = 7.98 years					

The operating expenses consists of chemical and energy consumption, sludge disposal, labor costs, and others, among which electrical energy consumption accounts for about 99% of operating expenses at 0.16 USD/kWh, the largest of all operational expenses, which is consistent with previous findings by Hashemi et al*.*^70,71^. Regarding chemical consumption, operating the MBR at the optimal conditions of 0.86 kg COD/m^3^d will postpone the membrane fouling. Further, each time TMP reached 0.4 bar, the membrane undergoes an automated physical cleaning intended to remove the cake layer from the membrane surface. Therefore, after fully fouling, the membrane module can be cleaned with disinfectants like sodium hypochlorite and citric acid for a relatively low cost as compared to other cleaning agents like ozone. This efficient chemical method of cleaning the membrane using 0.2% sodium hypochlorite solution can be carried out twice a year at a total cost of 2.7 USD/y. In addition, sludge disposal will be performed every four months at cost of 1.2 USD.

Because the MBR system is automated, close supervision is not required. As a result, the MBR is more cost-effective as the labor costs, or some of them, could be replaced by computational facilities, which opens the door to applying the MBR at larger and fuller scales. For that reason, the cost of labor for the technician in charge of follow-up and maintenance can be neglected. Finally, Based on the estimated capital and operational expenses, the overall cost of the pilot-scale MBR treatment for municipal wastewater at a flow rate of 20 m^3^/d is 54,818.3 USD.

From Table 3, the payback period should be 7.98 years under the profitability scenario that is based on reusing the treated municipal wastewater in the municipal treatment plants. Nicolaidis and Vyrides^32^ estimated the saving cost of reused wastewater at 1.13 Euro/1.0 m^3^, which is higher than our estimation, and they reported that the payback period could be only 6.0 years for pilot-scale MBR for treating wastewater. Similarly, when Tawfik et al.^34^ used a MBR for a treatment capacity of 30 m^3^/d of wastewater containing hazardous compounds, namely dioxane, they reported that the payback period in their study would be 6.6 years. This finding may be comparable to the current inquiry because both studies employed a similar estimate of the cost savings from using treated wastewater, which reached 0.96 USD/m^3^ in Tawfik et al.^34^ and 0.86 USD/m^3^ in this study. The influent properties in the aforementioned study would be similar to those of municipal wastewater because bacteria were adapted for a short period to a modest amount of dioxane. Therefore, the results in the two studies were comparable and the payback period did not change very much.

In conclusion, though biological treatments of wastewater using activated sludge are more cost-effective than other treatments, the addition of MBR would enhance their efficiency and cost-effectiveness even further^72^. The economic viability of the entire treatment process is significantly improved by such a combination. The MBR system is a great choice for countries expected to face water scarcity in the near future, like Egypt. This technology offers an excellent solution for the sector's economic viability as well as benefiting the environment since it provides establishments with the facility to close the water cycle.

## Conclusions

In the present investigation, the impact of OLR on MBR performance at a specified HRT 7 h and SRT 30 days was assessed. The information obtained led to the following conclusions:

An increase in OLR resulted in a correspondingly inverse rise in biomass concentration in the MBR. The MBR's COD, NH_4_–N, and BOD removal efficiency were at their maximum at the low OLR. The efficiency of nitrogen removal considerably decreased as OLR increased from 1.8 to 3.7 kgCOD/m^3^d, and the number of nitrifying bacteria also increased. When OLR was raised further to 3.7 kgCOD/m^3^d, nitrogen removal dropped. Further, the optical and SEMmicrographs displayed the discrepancy between the three operating systems. While OLR values of 0.86 and 1.8 kg COD/m^3^d display a moist mount of big, compact, solid, well-settling flocs, the OLR 3.7 kg COD/m^3^d system shows open flocs and loose flocs with inter-floc filament bridging. Accordingly, the disappearance of filamentous bacteria when MBR was run at OLR 0.86 and 1.8 kg COD/m^3^d provided more evident proof of the cause of the performance disparity between the three operating systems. According to the economic analysis of the MBR system, the payback period for using the treated wastewater will be 7.98 years for a planned flow rate of 20 m^3^/d, confirming the MBR's economic advantages in treating municipal wastewater. The outcomes from this study support the idea that using MBR technology to treat wastewater can boost system sustainability while using fewer chemicals and protecting environmental issues. Thus, treatment systems in Egypt could depend on a larger MBR scale in the future.

More in-depth studies should be done to address the drawbacks, such as energy consumption and membrane fouling, to lower costs and maximize performance as MBR demand in various sectors is anticipated to increase in the future. As well, to develop more efficient MBR technologies, attention should be given to the development of low-cost, high-performing membrane materials.

## Data Availability

All data generated or analysed during this study are included in this published article.
